# Area Selective Deposition of Metals from the Electrical Resistivity of the Substrate

**DOI:** 10.1021/acs.jpclett.1c00415

**Published:** 2021-04-22

**Authors:** Hama Nadhom, Robert Boyd, Polla Rouf, Daniel Lundin, Henrik Pedersen

**Affiliations:** Department of Physics, Chemistry and Biology, Linköping University, SE-58183 Linköping, Sweden

## Abstract

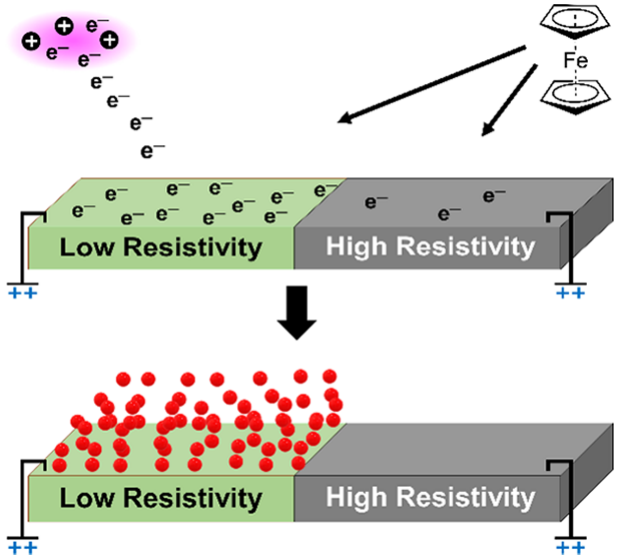

Area selective deposition (ASD) of films only on desired areas of the substrate opens for less complex fabrication of nanoscaled electronics. We show that a newly developed CVD method, where plasma electrons are used as the reducing agent in deposition of metallic thin films, is inherently area selective from the electrical resistivity of the substrate surface. When depositing iron with the new CVD method, no film is deposited on high-resistivity SiO_2_ surfaces whereas several hundred nanometers thick iron films are deposited on areas with low resistivity, obtained by adding a thin layer of silver on the SiO_2_ surface. On the basis of such a scheme, we show how to use the electric resistivity of the substrate surface as an extension of the ASD toolbox for metal-on-metal deposition.

Miniaturization of electronic components has increased the demand for precise and improved thin film deposition methods. For example, deposition only on desired areas would avoid the need for patterning and etching steps and allow for bottom-up fabrication of nanoscaled structures. This has motivated research into area selective deposition (ASD) by chemical vapor deposition (CVD) techniques.^1−3^ The selectivity in ASD is usually achieved by changing the surface chemistry of the area where film growth is either desired or not desired to control the adsorption of precursor molecules to only specific areas on the substrate. Different methods have been reported to enable ASD. Self-assembled monolayers of organic molecules are often used to block areas of the surface where no film growth is desired.^4^ Film nucleation can also be inhibited on certain areas on the surface by surface passivation using ion implantation of fluorocarbons^5^ or by poisoning the surface by inhibitors such as fluorine, hydrogen, or ammonia.^6,7^ ASD can also be achieved by utilizing different film growth rate or nucleation rates on different materials on the substrate.^8^ This approach can be further enhanced by adding etching steps to the deposition process.^9^

We recently reported a new CVD method for deposition of metallic films where the free electrons in a plasma are used as reducing agents.^10^ Because the method draws an electron current from the plasma to an electrically biased substrate, a conducting surface is needed to close the electric circuit allowing the electron current to flow from the plasma discharge to the bias power supply without any charge buildup. Therefore, metallic films could be deposited on electrically conducting silver substrates, while the deposition process was hampered on poorly conductive silicon and inactive on insulating silicon dioxide substrates. Here, we show how this inherent surface selectivity can be used to afford an area selective deposition of metallic thin films on only areas of a substrate surface that are electrically conducting.

Iron films were deposited from ferrocene, bis(cyclopentadienyl)Fe(II) (FeCp_2_), with plasma electrons as reducing agents on a 1 × 2 cm^2^ Si (100) substrate with a 300 nm thermally grown SiO_2_ layer, partially covered by a 40 nm thick layer of sputter-coated silver. The deposition system setup and the experimental procedures of our new CVD method are described elsewhere.^10^ Briefly, the depositions were performed in a vacuum chamber equipped with a hollow cathode plasma source located in the top lid of the vacuum chamber. Argon was used as working gas at a flow rate of 70 sccm through the titanium hollow cathode. The plasma discharge was maintained at 70 W in DC mode at a working gas pressure of 25 Pa. FeCp_2_ was supplied by evaporation at 70 °C, and the vapor was drawn into the deposition chamber by the chamber vacuum. The stainless-steel substrate holder (65 × 42 × 1 mm^3^) was placed in the precursor stream, upstream from the plasma source to allow precursors to adsorb on the substrate without entering the plasma bulk to minimize plasma chemical decomposition of the metal precursors. A DC bias voltage of 40 V, connected to the steel sample holder, was used to attract the plasma electrons to the substrate. Drawing an electronic current resulted in a slight heating of the substrate holder, and a temperature of 40 °C was measured by a thermocouple spot-welded to the backside of the substrate holder. This was regarded as the deposition temperature as no other heating was used. The substrates were electrically connected to the substrate holder by using silver paint on both the silver-coated and the SiO_2_ sides of the substrate. The deposition time was 60 s.

Cross-section scanning transmission electron microscopy (STEM) was used to determine the presence, thickness, and chemical structure of the deposited films. Prior to analysis, thin sections suitable for analysis by STEM were prepared by using the lift-out approach based on focused ion beam milling.^11^ All STEM analyses were performed by using a FEI Tecnai G2 TF 20 UT instrument operated at 200 kV. Images were collected with an annular detector spanning the range 80–260 mrad. Top-view scanning electron microscopy (SEM) was done by using an LEO 1550 Gemini SEM instrument with an acceleration voltage of 3 kV. We determined the composition and the elemental mapping of the deposited films layers by energy dispersive X-ray spectroscopy (EDS) using the same SEM system with an acceleration voltage of 20 kV.

X-ray photoelectron spectroscopy (XPS) was used to analyze the elemental composition and chemical bonding in the deposited films using monochromatic Al Kα X-rays. A charge neutralizer filament was used to compensate for the charge buildup effect. The conditions used for survey scans were as follows: energy range = 0–1200 eV, pass energy = 160 eV, step size = 0.1 eV, and X-ray spot size = 2 mm in diameter. A binding energy range of 20–40 eV (depending on the examined peak) was used for high-resolution spectra with a pass energy of 20 eV. Argon (0.5 keV) was used as the sputtering source. The C 1s peak with a value of 285 eV was used for calibration in all spectra. Gaussian–Laurentius (GL) functions and a Shirley background were used to fit the experimental XPS data.

Cross-section STEM analysis (Figure 1) shows that an ∼400 nm thick film is deposited on the Ag-coated areas of the SiO_2_ (resistivity ρ_Ag,bulk_ = 1.59 × 10^–8^ Ω·m)^12^ (Figure 1a), while no film can be seen on the bare SiO_2_ (resistivity ρ_Silica_ = 5 × 10^11^–10^14^ Ω·m)^12^ (Figure 1b). The deposited Fe film is relatively rough, with height variations of 100 nm and porous; the film appears more dense near the Ag layer. Compositional mapping by STEM-EDS (insets in Figures 1a and 1b) reveals that the film deposited on Ag-coated area is Fe-rich. Further analysis of the EDS data shows that the film also has a high O content (composition FeO_1.3_) with trace amounts of Ti and C. The presence of Ti is most likely due to sputtering of the titanium hollow cathode, and the presence of C is in part due to contamination during preparation and analysis. This indicates that the analyzed films have a high concentration of iron oxide due to the porous nature of the film (Figure 1), a significant portion of which could have been formed when the newly revealed Fe was exposed to air. The selected area electron diffraction of the iron film shows no diffraction spots, and HRTEM analysis of the film could not resolve any diffraction fringes (data not shown), both of which indicate that the film is amorphous when analyzed. The TEM analysis of the substrate areas not coated by Ag shows no film growth, and no iron could be detected by EDS.

**Figure 1:**
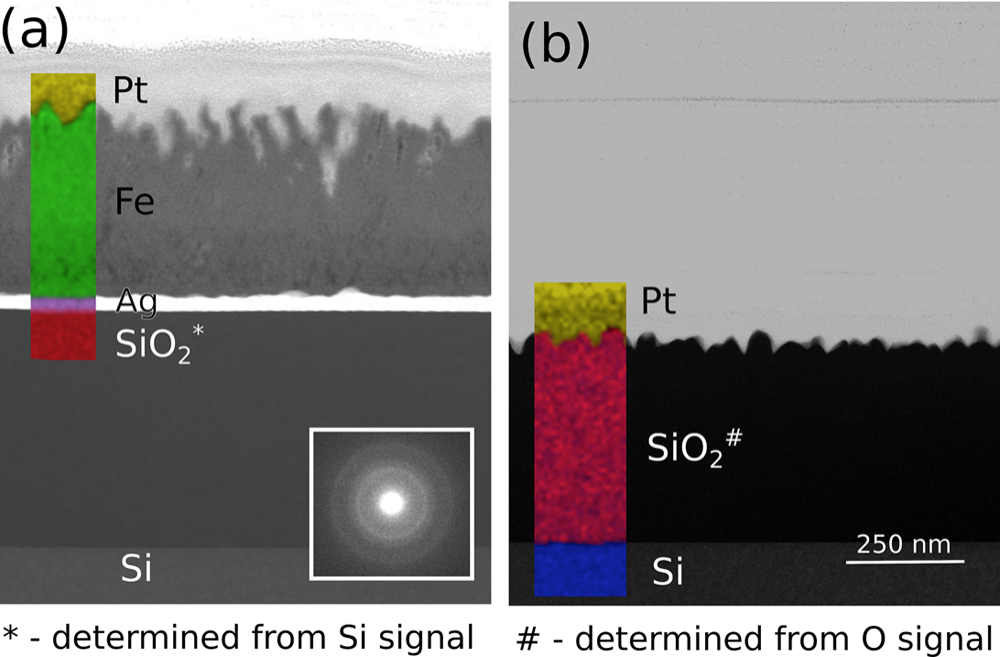
Cross-sectional scanning transmission electron micrographs on the substrate regions with (a) and without (b) Ag coating. Insets show the compositional maps, which were derived directly from the STEM-EDS data. SAED of the deposited films are given as insets.

From the TEM analysis it is seen that both the deposited iron and the uncoated silicon oxide substrate material have a very rough morphology with height variations of 50 nm. As we cannot deposit an oxidation-blocking cap layer after the metal deposition in our deposition system, we deposit thicker Fe films to avoid complete oxidation of the very oxyphilic Fe film. This meant that we used much longer processing times than needed for any relevant application in nanometer-scaled electronic applications where typical film thicknesses of only a few tens of nanometers are needed. From Figure 1 it is seen that the first ∼100 nm close to the Ag interface is significantly denser as compared to the top surface. This region is also more than a relevant film thickness for electronics applications. The longer deposition meant that whatever mechanism causing the increased surface roughness on the SiO_2_ was exaggerated. Thus, deposition of thinner Fe films is expected to significantly reduce the surface roughness on the SiO_2_. We speculate that the roughening mechanism is somewhat similar to preferential sputtering of insulating layers in asymmetric bipolar DC-sputtering discharges, as described by Sellers.^13^ The insulating SiO_2_ forms a parasitic capacitor, which leads to the plasma-facing surface of the insulator to be charged toward −40 V when applying +40 V to the sample holder. This opens for positive ion acceleration toward the SiO_2_ surface and thereby etching of the top surface. There is also the possibility that we build up voltage on the compound which could cause a breakdown of these parasitic capacitors,^14^ also resulting in increased surface roughness.

Top view SEM with EDS elemental mapping of the interface between the Ag-coated and bare SiO_2_ surfaces (Figure 2) further show the area selectivity of the deposition process with Fe detected only on the Ag-coated area.

**Figure 2:**
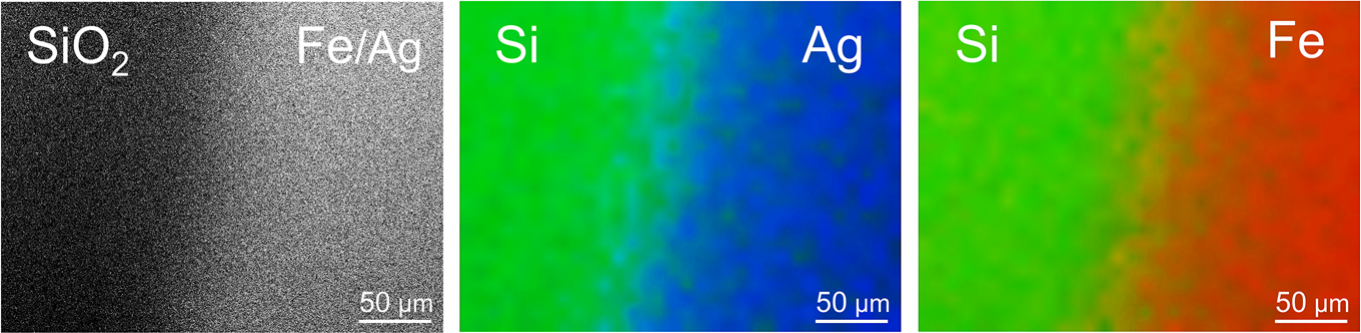
Top-view scanning electron micrographs on the interface between the Ag-coated and bare SiO_2_ surface regions. The elemental mapping by energy dispersive X-ray spectroscopy shows that the Fe is deposited only on the Ag-coated surface region.

High-resolution XPS of the Fe spectral region recorded from the Si/SiO_2_ substrate areas with and without Ag coating (Figure 3) shows that the films deposited on the Ag-coated areas consist of iron, confirming the EDS analysis (Figures 1 and 2). The XPS analysis shows that the films deposited on the Ag-coated areas are mixed iron oxides in the as-deposited, untreated samples. The Fe 2p region (Figure 3a) shows peaks at 709.5–711.5 eV, corresponding to Fe–O.^15^ It should be noted that these samples were subjected to air prior to being loaded into the XPS chamber, and given the oxyphilic nature of iron, surface oxidation is expected. Film deposition was also done in medium vacuum, meaning that low levels of oxygen exposure are to be expected during the deposition.^16^ The films where therefore sputter cleaned in the XPS chamber. After 1800 s sputtering, shoulder peaks at 706.9 and 720.1 eV (Δ13.2 eV) corresponding to zerovalent Fe 2p_3/2_ and Fe 2p_1/2_,^15,17^ respectively, can be seen (Figure 3a). After further sputtering to a total sputter time of 3000 s these peaks dominate the spectra. The composition analysis by XPS of sputtered clean films deposited on the Ag-coated SiO_2_ shows 40 at. % Fe, 19.5 at. % C, 35 at. % O, 2.6 at. % N, and 2.9 at. % Ti. In contrast, XPS analysis of the Si/SiO_2_ substrate areas not coated by Ag shows very similar, albeit weaker, peaks, ascribed to iron oxides, in the as-deposited samples (Figure 3b). As these films were sputter cleaned for 1800 s, no change in peak position is observed, and the peaks in the iron spectral region vanish after 3000 s sputtering on the SiO_2_ substrate, indicating a very thin iron oxide which could not be detected in the STEM analysis (Figure 1b). This shows that the amount of Fe deposited on the SiO_2_ substrate is very low compare to films deposited on the Ag-coated part of the substrate.

**Figure 3:**
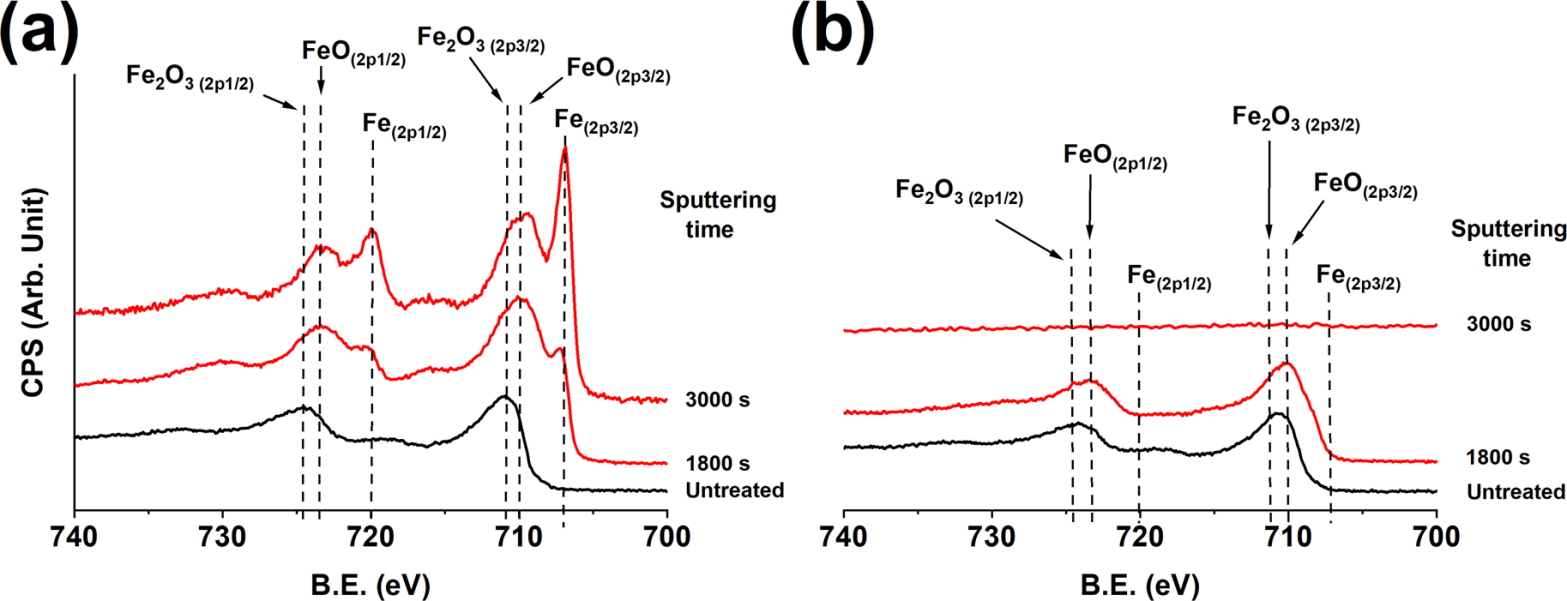
High-resolution XPS spectra, showing the iron spectral region of deposited films on (a) the substrate regions with Ag-coated SiO_2_ and (b) without Ag-coated SiO_2_.

Our experimental results show that Fe has been selectively deposited on Ag, adding to the previous literature on ASD of Fe on metals.^18^ Our results also suggest that the electrical resistivity of the surface determines the ability to deposit metallic films by this CVD method and that the CVD method is inherently area selective from the surface electrical resistivity. This inherent area selectivity of the new CVD process, where plasma electrons are used as reducing agents, does not depend on any thermodynamic or kinetic factors, as in other inherent ASD processes.^19^ Instead, creating a low-resistivity path for the electron flux from the plasma discharge to the substrate bias supply via the substrate surface is the prerequisite for the deposition chemistry in this new CVD process. We believe that this opens for exciting new possibilities for metal deposition selectively on metals.
